# The CD28-Transmembrane Domain Mediates Chimeric Antigen Receptor Heterodimerization With CD28

**DOI:** 10.3389/fimmu.2021.639818

**Published:** 2021-03-23

**Authors:** Yannick D. Muller, Duy P. Nguyen, Leonardo M. R. Ferreira, Patrick Ho, Caroline Raffin, Roxxana Valeria Beltran Valencia, Zion Congrave-Wilson, Theodore L. Roth, Justin Eyquem, Frederic Van Gool, Alexander Marson, Laurent Perez, James A. Wells, Jeffrey A. Bluestone, Qizhi Tang

**Affiliations:** ^1^Department of Surgery, University of California, San Francisco, San Francisco, CA, United States; ^2^Diabetes Center, University of California, San Francisco, San Francisco, CA, United States; ^3^Department of Medicine, Service Immunologie et Allergie, Centre Hospitalier Universitaire Vaudois (CHUV), Lausanne, Switzerland; ^4^Department of Pharmaceutical Chemistry, University of California, San Francisco, San Francisco, CA, United States; ^5^Sean N. Parker Autoimmune Research Laboratory, University of California, San Francisco, San Francisco, CA, United States; ^6^Department of Medicine, University of California, San Francisco, San Francisco, CA, United States; ^7^Gladstone-UCSF Institute of Genomic Immunology, Gladstone Institutes, San Francisco, CA, United States; ^8^Department of Cellular and Molecular Pharmacology, University of California, San Francisco, San Francisco, CA, United States

**Keywords:** chimeric antigen receptor, CAR T cell, CD28, transmembrane domain, hinge domain, heterodimerization, dimer, CAR

## Abstract

Anti-CD19 chimeric antigen receptor (CD19-CAR)-engineered T cells are approved therapeutics for malignancies. The impact of the hinge domain (HD) and the transmembrane domain (TMD) between the extracellular antigen-targeting CARs and the intracellular signaling modalities of CARs has not been systemically studied. In this study, a series of 19-CARs differing only by their HD (CD8, CD28, or IgG_4_) and TMD (CD8 or CD28) was generated. CARs containing a CD28-TMD, but not a CD8-TMD, formed heterodimers with the endogenous CD28 in human T cells, as shown by co-immunoprecipitation and CAR-dependent proliferation of anti-CD28 stimulation. This dimerization was dependent on polar amino acids in the CD28-TMD and was more efficient with CARs containing CD28 or CD8 HD than IgG_4_-HD. The CD28-CAR heterodimers did not respond to CD80 and CD86 stimulation but had a significantly reduced CD28 cell-surface expression. These data unveiled a fundamental difference between CD28-TMD and CD8-TMD and indicated that CD28-TMD can modulate CAR T-cell activities by engaging endogenous partners.

## Introduction

Chimeric antigen receptor (CAR)-engineered T cells are emerging as promising therapies for otherwise untreatable diseases (1). The United States Food and Drug Administration (FDA) has approved two anti-CD19 CAR (19-CAR) T-cell products, namely tisagenlecleucel (CTL-019, KYMRIAH, Novartis Pharmaceuticals Corp.) and axicabtagene ciloleucel (KTE-19, YESCARTA, Kite Pharma, Inc.), for the treatment of acute lymphocytic leukemia and relapsed/refractory large B-cell lymphoma. A third CAR-T product, lisocabtagene maraleucel (JCAR-17, LISO-CEL, Bristol-Myers Squibb), is currently under review by the FDA for adults with relapsed/refractory large B-cell lymphoma. The success of these CAR-T products can be attributed to their antigen specificity, all conferred by the single-chain variable fragment (scFv) of the anti-CD19 antibody clone FMC63 and their intracellular signaling domains (ICDs), namely 28**ζ** for KTE-19 (2–4) and 4-1BB**ζ** for CTL-019 (5–8), and JCAR-17 (9). It is worth noting that these products differ in their hinge domain (HD) and transmembrane domain (TMD), that is, CD28-HD/TMD for KTE-19, CD8-HD/TMD for CTL-019, and IgG_4_-HD/CD28-TMD for JCAR-17.

In earlier iterations of CAR designs, CD28- and CD8-TMDs were chosen because they are considered to be inert when compared to the CD3z-derived TMD that mediated the association of the CAR with the endogenous T-cell receptor (TCR)/CD3 complexes (10). Emerging evidence, however, suggests potential contributions of the HD and the TMD to the function of CAR-T cells. The group of June first observed unexpectedly sustained proliferation after a single *in-vitro* stimulation of CD28-HD/TMD-based-CAR T cells, but not CD8-HD/TMD-based-CAR T cells, directed against mesothelin (11). Majzner et al. (12) demonstrated that replacing a CD8-HD/TMD with a CD28-HD/TMD lowers the threshold for CAR activation to CD19 in an ICD-independent fashion. These results corroborate the findings reported by Kochenderfer et al. and show that T cells with CD28-HD/TMD-containing CARs secrete higher levels of interferon-γ upon CAR stimulation (13, 14).

The mechanisms underlying the differences between CD8-HD/TMD and CD28-HD/TMD domains remain to be defined (15). In the present study, the impact of CD28-TMD on 19-CARs in human T cells was investigated, and it was discovered that CD28-TMD mediated a transmembrane domain-dependent heterodimeric association of the CAR with the endogenous CD28 receptor.

## Materials and Methods

### Human T-Cell Isolation

Human blood from deidentified normal donors was purchased from STEMCELL Technologies (Vancouver, Canada), which collected and distributed de-identified human blood products with consent forms, and according to the protocols, approved by the Institutional Review Board (IRB). Peripheral blood mononuclear cells were isolated by Ficoll density gradient centrifugation, and T cells were further enriched using the EasySep Human T Cell Isolation Kit (STEMCELL Technologies) as per the instructions of the manufacturer. Enriched T cells or CD4^+^CD127^+^CD25^low^ conventional T cells purified by fluorescence-activated cell sorting (FACS) were used for experiments. Cells were either used fresh or cryopreserved in fetal calf serum (FCS) with 10% DMSO. Frozen cells, when used, were thawed and cultured overnight in 300 IU/ml of IL-2 before editing and cell activation.

### Genome Editing Using Ribonucleoprotein Complex

Ribonucleoprotein complexes (RNPs) were made by mixing CRISPR RNAs (crRNAs) and trans-activating crRNAs (tracrRNA, Integrated DNA Technologies, Coralville, IA) with recombinant Cas9 protein (QB3 Macrolab, UC Berkley, CA) as previously described in a study by Roth et al. (16). Guide RNA sequences used for gene editing were as follows: (1) T-cell receptor β chain constant region (TRBC): CCCACCAGCTCAGCTCCACG; (2) CD19: CGAGGAACCTCTAGTGGTGA; and (3) CD28: TTCAGGTTTACTCAAAAACG. Lyophilized RNAs were resuspended at 160 μM in 10 mM Tris-HCl with 150 mM KCl and stored in aliquots at −80°C. On the day of electroporation, crRNA and tracrRNA aliquots were thawed and mixed at a 1:1 volume and annealed for 30 min at 37°C. The resulting 80 μM guide RNA complex was mixed at 37°C with Cas9 Nucleic Localization Signal (NLS) at a 2:1 gRNA to Cas9 molar ratio for another 15 min. The resulting RNP was used for genome editing. About 1 × 10^6^ T cells were mixed with appropriate RNP and electroporated using a Lonza 4D 96-well electroporation system (pulse code EH115) to delete *TCR* or *CD28* genes. For generating CD19^−^ variants of Raji cells, Raji cells (ATCC® CCL-86™, Manassas, VA) were electroporated (pulse code EH140) with RNP targeting *CD19*, and the CD19- negative fraction was purified by FACS after culturing the cells for more than 1 week.

### Gene Editing of Human T Cells

CD4^+^ T cells were gene-edited before stimulation with anti-CD3/CD28 beads (Dynabeads Human T-Activator CD3/CD28, Thermo Fisher Scientific, Waltham, MA). Cells were cultured in RPMI supplemented with 10% FCS and 300 IU/ml of IL-2 (Prometheus laboratories, Nestle Health Science, Lausanne Switzerland) for the first 2 days, and then, the concentration of IL-2 was reduced to 30 IU/ml for CD4^+^ T cells and to 100 IU/ml for bulk T cells. Lentiviruses encoding anti-CD19-I_4_-28-4-1BB**ζ**-T2A-EGFRt and anti-CD19-I_4_-28-28**ζ**-T2A-EGFRt were provided by Juno Therapeutics (Bristol-Myers Squibb, New York, NY). Other lentiviral constructs, present in Figure 2A, were cloned into the pCDH-EF1-FHC vector (Addgene plasmid #64874, Watertown, MA) as previously described by Hill et al. (17). Later, genes encoding CAR constructs were purchased from gBlocks™ Gene Fragments (Integrated DNA Technologies) (17, 18) and amplified by PCR and cloned into the pCDH vector using In-Fusion Cloning Tools (Takara Bio, Kusatsu, Japan). Sequences for all clones used in subsequent experiments were confirmed by sequencing. Transduction was performed on day 2 after CD4^+^ T cell activation at a multiplicity of infection of one by spinoculation (1,200 g, 30 min, 30°C) in a medium supplemented with 10% FCS and 0.1 mg/ml of protamine. For AAV production, 30 mg of helper plasmid pDGM6 (a kind gift from YY Chen, University of California, Los Angeles), 40 mg of pAAV helper, and 15 nmol PEI were utilized. AAV6 vector production was carried out by iodixanol gradient purification. After ultracentrifugation, the AAVs were extracted by puncture and further concentrated using a 50 ml Amicon column (Millipore Sigma Burlington, MA) and directly titrated on primary human T cells.

### *In vitro* Activation of Gene-Edited CAR T Cells

For some experiments, cells were restimulated on day 9 after primary stimulation without separating edited and transduced cells. For proliferation assays, the cell mixtures were stained with 2.5 μM carboxyfluorescein diacetate succinimidyl ester (CFDA SE, ThermoFisher, referred to as CFSE) before restimulation with anti-CD3/CD28 beads. For other experiments, cells were separated by FACS on day 9 to purify CD3^+^ and CD3^−^ T cells with or without CAR. For assessing CD25^+^CD71^+^ upregulation, purified CAR T cells were stimulated with parental CD19^+^ Raji cells or CD19^deficient^ Raji cells for 2 days. In some cultures, CTLA-4 Ig (provided by Dr. Vincenti, UCSF) was added at a concentration of 13.5 μg/ml. For measurements of proliferation, purified cells were stimulated with soluble anti-CD28 (clone CD28.2, 1 μg/ml, BD Pharmigen), plate-bound anti-CD28 (clone CD28.2, 10 μg/ml), or soluble anti-CD3 (clone HIT3α 2 μg/mL. BD Pharmigen). After 48 h, a portion of the supernatant was collected and analyzed for cytokine secretion using multiplexed Luminex (Eve Technologies, Calgary, Canada). The cells were then pulsed with 0.5 μCi of ^3^H thymidine and cultured for another 16–18 h before harvesting to determine the level of ^3^H thymidine incorporation using a scintillation counter.

### Flow Cytometry

The following antibodies were used for phenotyping and proliferation assays: anti-CD3-PE/Cy7 (clone SK7, BioLegend, San Diego, CA), anti-CD4-PerCP (clone SK3, BD Pharmigen, San Jose, CA), anti-CD4 A700 (clone RPA T4, BioLegend), anti-CD19 APC (clone HIB19, BD Pharmigen), anti-CD25 APC (clone 2A3, BD Pharmigen), anti-CD71 FITC (clone CY1G4, BioLegend), anti-Myc FITC or APC (clone 9B11, Cell Signaling, Danvers, MA), anti-FMC19 idiotype APC (Juno Therapeutics), anti-EGFRt PE (Juno Therapeutics), anti-CD28 APC (clone 28.2, Biolegend), and CD8 APC-Cy7 (clone SK1, BioLegend). DAPI (ThermoFisher, Waltham, MA) was used to stain dead cells for exclusion during analysis. Flow cytometric analyses were performed on an LSR II Flow Cytometer System (BD Biosciences). Fluorescence-activated cell sorting was performed on an FACSAria III Cell Sorter (BD Biosciences). All flow cytometry data were analyzed using the FlowJo software (Tree Star, Ashland, OR).

### Immunoprecipitation

FACS-purified CD3^−^CAR^+^ or CD3^−^CAR^−^ CD4^+^ T cells (8 × 10^6^ each) were lysed in Pierce™ IP Lysis Buffer (ThermoFisher) supplemented with cOmplete Protease Inhibitor Cocktail (Roche, Basel, Switzerland) for 30 min using a vertical rotator. Cell lysis was completed by briefly sonicating cells using a Q500 sonicator (QSonica, Newtown, CT). Pierce^TM^ anti-c-Myc magnetic beads (clone 9E10, ThermoFisher) were used for immunoprecipitation of the CAR. Alternatively, rabbit anti-human CD28 (clone D2Z4E, Cell Signaling) followed by anti-rabbit IgG Pierce^TM^ protein A/G magnetic beads (ThermoFisher Scientific) were used for CD28 immunoprecipitation of the cell lysate according to the instructions of the manufacturer.

### Western Blotting

Equal masses of protein lysate or equal volumes of immunoprecipitation eluents were loaded into NuPAGE 4–12% Bis-Tris, 1.0 mm gels (ThermoFisher Scientific). After electrophoresis, proteins were transferred onto PVDF membranes (ThermoFisher Scientific) using an iBlot 2 Dry Blotting System. After blocking with Tris-buffered saline with 0.1% Tween-20 and 5% bovine serum albumin (TBSTB), membranes were stained with primary and secondary antibodies diluted in TBSTB. The following antibodies used were mouse anti-Myc (clone 9B11, Cell Signaling), rabbit anti-CD28 (clone D2Z4E, Cell Signaling), HRP-conjugated anti-mouse IgG (Cell Signaling), and HRP-conjugated anti-rabbit IgG (Cell Signaling).

### Three-Dimensional Model Prediction and Validation

Structural modeling of the different CARs was performed using Iterative Threading ASSembly Refinement (I-TASSER) software (19). Amino acid corresponding to the scFv was modeled on the UCHT1 scFv template (PDB ID code 1XIW) (20). The HD coordinates were recovered from the crystal structure of the pembrolizumab template (PDB ID code 5DK3) (21) and the crystal structure of human CD28 (PDB ID code 1YJD) (22) for IgG4 and CD28, respectively. Modeling of the CD8-HD was performed using the Rosetta protein modeling suite (23). Structures were assembled with PyMOL (*Sc*hrodinger, LLC). Models were further evaluated with MolProbity software (24).

## Results

### Generation of 19-CAR T Cells With Various HDs and TMDs

**Figure 1:**
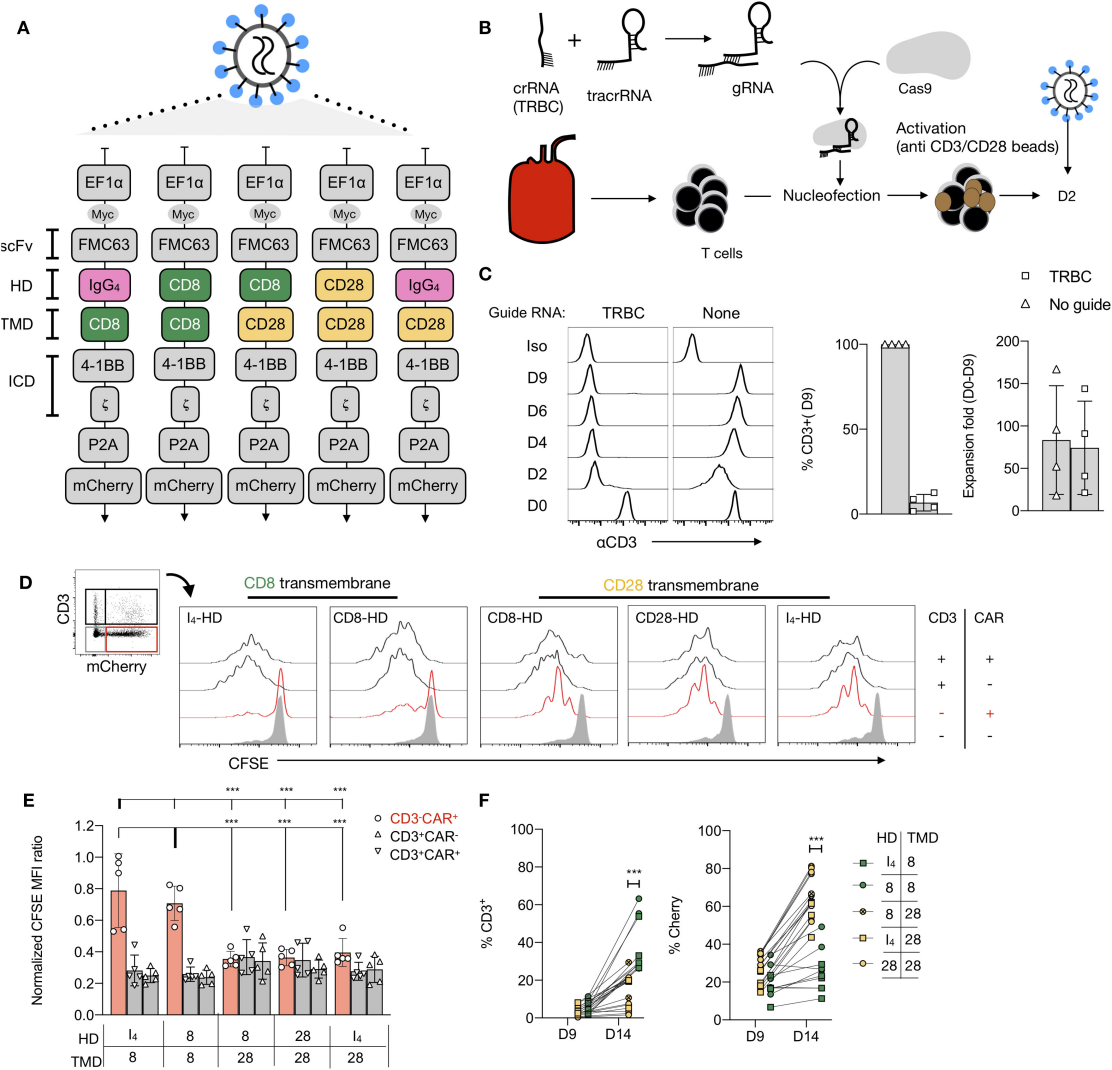
Anti-CD28 stimulation of CD19-chimeric antigen receptors (CAR) T cells is TMD dependent. **(A)** Designs of five CAR against CD19 bearing a 4-1BB costimulatory domain and differing by their hinge domain (HD) and their transmembrane domain (TMD). **(B)** Fluorescence-activated cell sorting (FACS) -sorted CD4^+^CD127^+^CD25^low^ T cells were electroporated with a CRISPR-Cas9 ribonucleoprotein complex (RNP) targeting the constant region of the TCR β chain gene (TRBC), followed by stimulation with anti-CD3/CD28 beads (1:1 ratio). **(C)** Representative results of flow cytometric analysis of the CD3 expression over time of cells electroporated with or without RNP. Percentages of residual CD3^+^ population and fold-expansion after 9 days of culture of CD4^+^ T cells electroporated with or without RNPs targeting TRBC are shown. Results from four independent experiments. **(D)** A representative example of carboxyfluorescein diacetate succinimidyl ester (CFSE) dilution of a mixed population of CD3^+/−^CAR^+/−^ T restimulated with anti-CD3/28 beads. **(E)** The normalized CFSE mean fluorescence intensity (MFI) ratio for CD3^−^mCherry^+^, CD3^+^mCherry^−^, and CD3^+^mCherry^+^ cells was calculated by dividing CFSE MFI of these populations with the MFI of the CD3^−^mCherry^−^ cells in the same culture. Two-way ANOVA was used for statistical analysis (bold line set as reference). **(F)** Percentages of CD3^+^ and mCherry^+^ cells before and 5 days after restimulation of edited T cells with anti-CD3/CD28 beads. The Unpaired *t*-test was performed by comparing CD8-TMD and CD28-TMD-containing CARs on D14. For **(E,F)**, the results shown are a summary of two independent experiments using T cells from five unrelated donors for each construct. ***p < 0.001.

To investigate the role of CAR TMD, we first generated a panel of 19-CARs differing only by their HD (CD8, CD28, or IgG_4_) and their TMD (CD8 vs. CD28), all of which have been used to engineer CAR T cells for clinical applications (Figure 1A, Supplementary Figure 1). Each CAR was designed with an MYC tag on the N-terminus of the scFv and a mCherry reporter (Figure 1A). For most experiments, we selected 4-1BB as a costimulatory domain in the ICD to avoid potential interactions with the endogenous CD28. Furthermore, we disrupted the *TRBC* locus using CRISPR/Cas9 to prevent any potential confounding influence by the endogenous TCR (Figure 1B). The *TRBC* gene-disrupted human T cells retained the cell surface expression of TCR/CD3 proteins for a few days after editing and could thus be activated with anti-CD3/CD28 beads. Edited CD4^+^ T cells were transduced with various lentiviral CAR constructs by spinoculation 2 days after activation. On day 9, after stimulation, 87–98% of the cells were found to CD3-negative, demonstrating successful TCR deletion in the majority of the cells (Figure 1C). Comparable transduction efficiencies were observed across the different CAR constructs, as assessed by the mCherry expression and all CAR T cells responded to CD19 restimulation (Supplementary Figures 2A–C).

### CAR T Cell Proliferation in Response to Anti-CD28 Stimulation

Restimulation of TCR-edited CAR-transduced T cells, containing a mixed population of CD3^+/−^ and CAR^+/−^ cells, with anti-CD3/CD28 beads on day 9, resulted in the expansion of CD3^+^ T cells that escaped TCR deletion (Figures 1D,E). However, TCR-deficient CD3^−^CAR^+^ T cells with CARs containing a CD28-TMD, but not CD8-TMD, also proliferated. Consequently, CAR^+^ T cells with a CD28-TMD, but not a CD8-TMD, were enriched at the end of the 5-day restimulation (Figure 1F). The lack of proliferation of CD8-TMD-containing CAR T cells showed that expansion was not a consequence of bystander effects, such as IL-2 production by the CD3^+^CAR^+^ T cells in the same culture. To determine if this is unique to CARs with 4-1BB-ICD, the experiment was repeated using CARs with a CD28-ICD, and a similar pattern of proliferation and enrichment of CD3^−^CAR^+^ T cells after anti-CD3/28 bead restimulation was observed (Supplementary Figures 3A–D).

**Figure 2:**
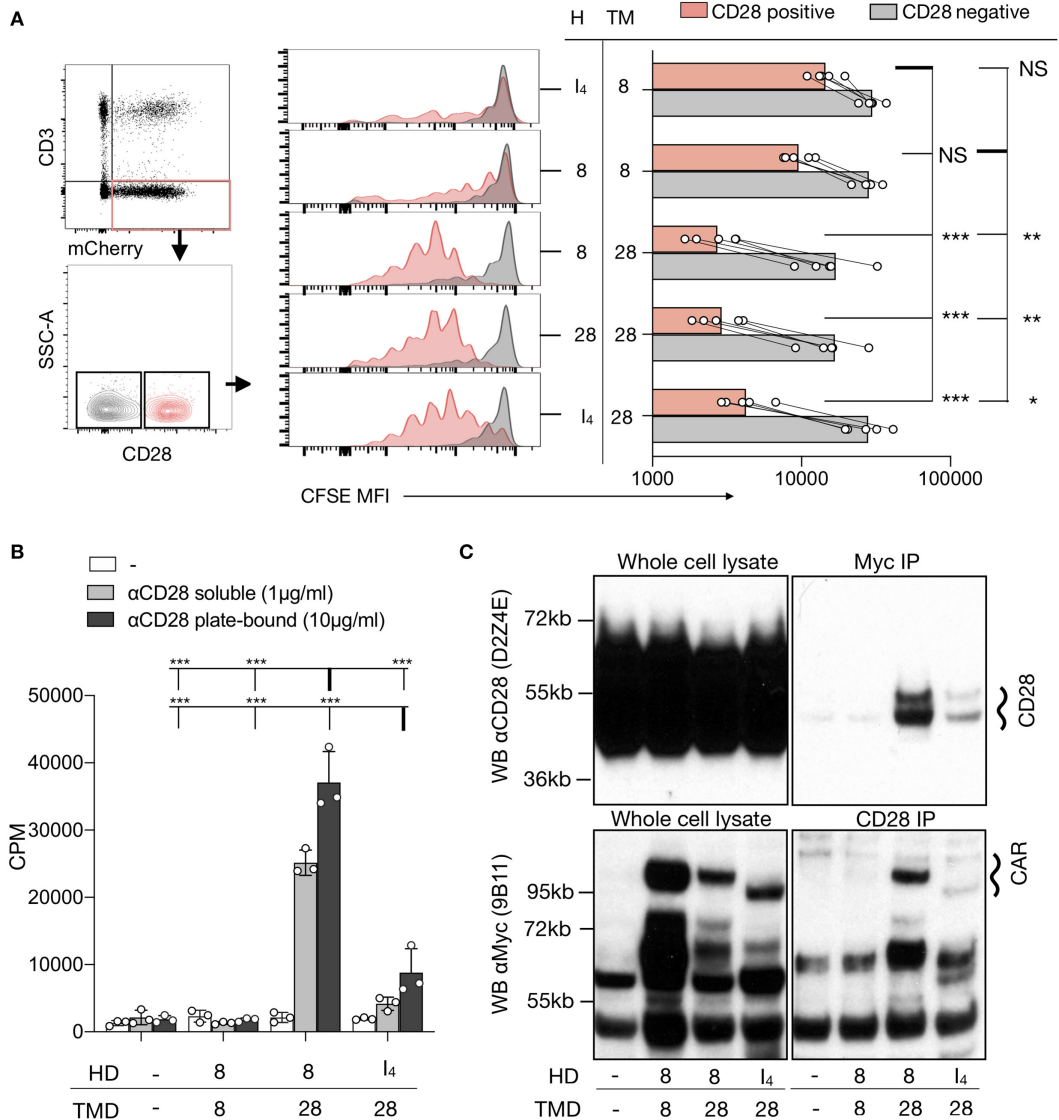
CD28-TMD-containing CARs interact with CD28. **(A)** A mixture of CFSE-labeled CD4^+^ T cells with or without CD3, CD28, and CAR expression. CFSE MFI of five independent donors in two independent experiments is reported. The one-way ANOVA was used for statistical analysis. **(B)** Proliferation of purified CD3^−^CAR^+^ CD4^+^ T cells in response to plate-bound or soluble anti-CD28 stimulation. Results are representative of three independent experiments. The two-way ANOVA was used for statistical analysis. **(C)** CD28 or the Myc-tag of CD3^−^CAR^+^ T cells were immunoprecipitated. Western blot analysis of the input (5% of the whole cell lysate) as well as of the precipitated cells was performed using anti-CD28 (clone D2Z4E) and anti-Myc (clone 9B11). Results are representative of two to three independent experiments for each condition. *p < 0.05, **p < 0.01, ***p < 0.001 Counts per minute (CPM). Not Statistically Significant (NS).

Since the CD3^−^CAR^+^ T cells had no TCR expression on the cell surface, the proliferation was likely stimulated by the anti-CD28 component of the anti-CD3/28 beads. To verify the need for the endogenous CD28 receptor for proliferation in response to anti-CD3/CD28 beads, both the *CD28* and *TRBC* genes were deleted in T cells before activation and lentiviral CAR transduction (Figure 2A). CD3^−^CAR^+^CD28^+^ T cells expressing CARs containing a CD28-TMD, but not a CD8-TMD, proliferated in response to anti-CD3/28 beads. The deletion of CD28 abrogated the ability of CD28-TMD-containing CAR T cells to proliferate in response to anti-CD3/CD28 beads, demonstrating that anti-CD28-induced activation was dependent on endogenous CD28 (Figure 2A). These results excluded the possibility that anti-CD3/CD28 beads directly bind to the CAR.

To further confirm that CD3^−^ T cells with a CD28-TMD-containing CAR could respond to anti-CD28 stimulation in the absence of other cells in the culture, we FACS-purified CD3^−^CAR^+^ cells before restimulation with plate-bound or soluble anti-CD28 antibodies (clone CD28.2). For these experiments, we excluded CD28-HD containing CARs to avoid potential interaction mediated by the CD28-HD. The results confirmed that CAR T cells engineered with a CD28-TMD, but not a CD8-TMD, proliferated in response to anti-CD28 alone (Figure 2B). The proliferative response induced by anti-CD28 alone in CD3^−^CAR^+^ T cells was similar in CAR T cells with a 4-1BB or a CD28 costimulatory domain in their ICD (Supplementary Figure 4A). Moreover, anti-CD28 induced the secretion of multiple cytokines by CD3^−^CAR^+^ T cells but not by CD3^+^CAR^−^ or CD3^−^CAR^−^ control cells (Supplementary Figure 4B). Collectively, these results show that CD28-TMD containing CARs can be activated by anti-CD28 without antigen recognition by the CAR or the TCR.

### CD28 and CAR Interaction

The results discussed earlier, together with recent reports of phosphorylation of endogenous CD28 upon CAR stimulation (all with a CD28-TMD domain) (25, 26), suggest interactions between CD28 and CD28-TMD-containing CARs. To directly determine if CD28-TMD-containing CAR and CD28 can physically interact, we performed co-immunoprecipitation experiments. CD28-TMD-containing, but not CD8-TMD-containing, CARs co-immunoprecipitated with endogenous CD28. Conversely, endogenous CD28 co-immunoprecipitated with CD28-TMD-containing, but not CD8-TMD-containing, CARs demonstrated that the CD28-TMD of the CAR interacted with the endogenous CD28 receptor (Figure 2C). CD8-HD/CD28-TMD CARs and CD28 co-immunoprecipitated more efficiently when compared to the IgG_4_-HD-CD28-TMD construct, which is consistent with improved proliferation observed with CD8-HD/CD28-TMD CAR upon anti-CD28 stimulation (Figure 2B). Because of the difficulties in expanding CD3^−^CD28^−^ CAR T cells and the unlikelihood that anti-CD28 mAb directly binds to the CD28 TMD, we did not perform the purified CAR T-cell proliferation and immunoprecipitation studies with CD28^−^ T cells.

### Residues in CD28 TMD Involved in CD28-CAR Heterodimerization

**Figure 3:**
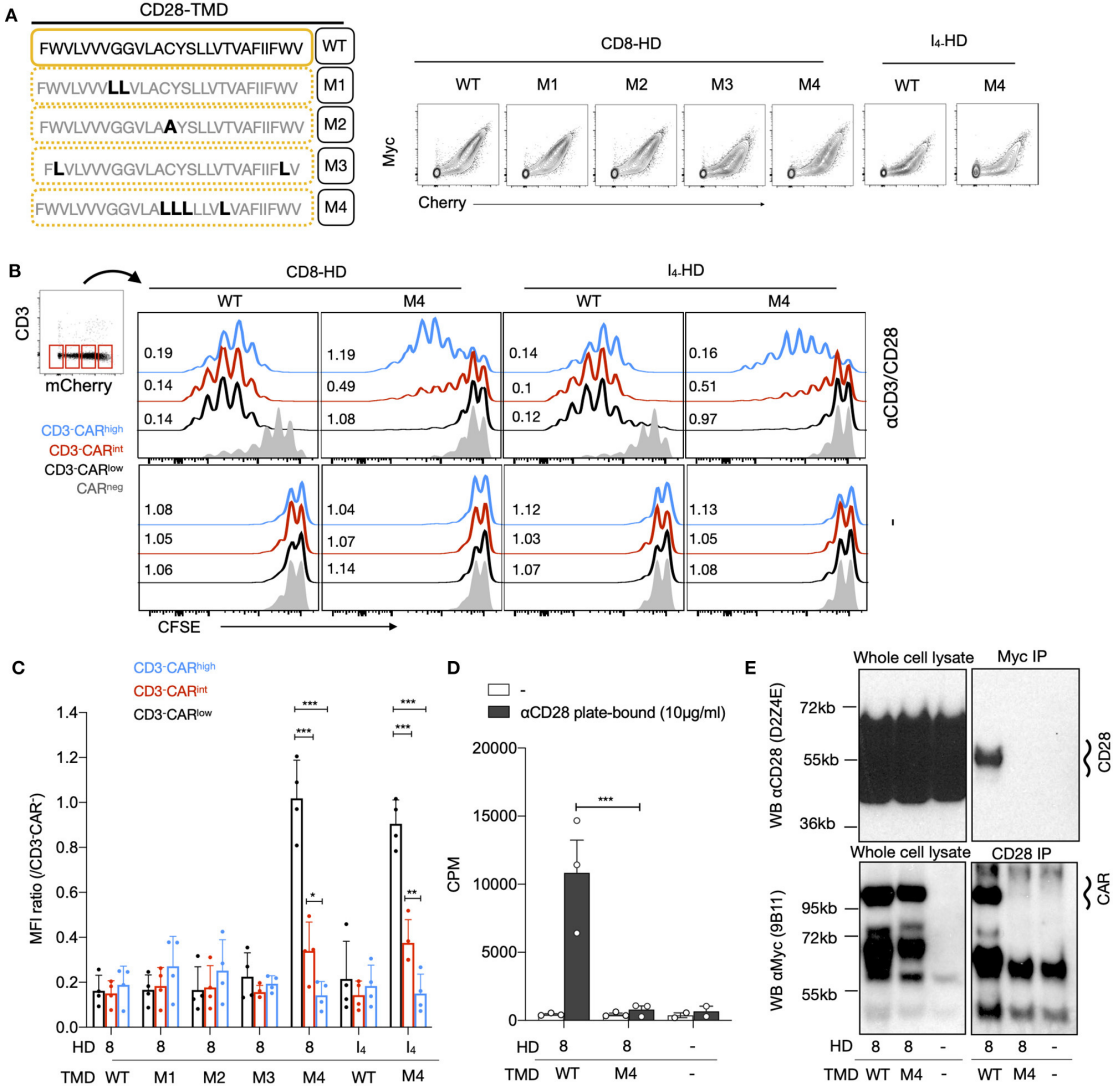
The dimerization of the CD28-TMD depends on a core of four amino acids. **(A)** Diagram representing the amino acid sequence of the wild type and four mutants of the CD28-TMD. A representative example of MYC and the mCherry expression for each mutant is shown. **(B)** A representative example of CFSE dilution of a mixed population of CD3^+/−^CAR^+/−^ T cells restimulated with anti-CD3/CD28 beads and unstimulated conditions. Representative CFSE MFI ratios (/CAR negative CD4 T cells) for low, intermediate, and high CAR expression with or without stimulation are shown **(C)** The normalized CFSE MFI ratio for CD3-CAR^low/int/high^ was calculated by dividing the MFI of each of these population with the MFI of CD3^−^mCherry^−^ cells within the same culture. A summary of results using T cells from four unrelated donors in two independent experiments is shown. **(D)** Proliferation of purified CD3^−^CAR^+^ T cells in response to plate-bound anti-CD28 stimulation. Results represent the mean of three independent experiments. **(E)** CD28 or the Myc-tag of CD3^−^CAR^+^ T cells were immunoprecipitated. Western blot analysis of the input (5% of the whole cell lysate) as well as of the precipitated was performed using anti-CD28 (clone D2Z4E) and anti-Myc (clone 9B11). Results are representative of two independent experiments for each condition. The two-way ANOVA was used for statistical analysis. *p < 0.05, **p < 0.01, ***p < 0.001.

Next, we generated a series of CD28-TMD CAR mutants to determine the molecular basis of the CAR–CD28 interaction. We first mutated the two glycines, G160L and G161L (M1), that may function as part of a glycine-zipper motif, a process known to control TMD dimerization (27). The second mutation replaced the C165 cysteine with alanine, as cysteine can form disulfide bonds (M2). The third (M3) mutations were made on two bulky hydrophobic tryptophans at the border of the TMD (W154L and W179L), and the fourth mutation (M4) targeted four amino-acid residues (C165L, Y166L, S167L, and T171L) present at the core of the TMD, as cysteine could form a disulfide bond and others may form hydrogen bond (Figure 3A). All CARs with TMD mutants were readily expressed on the cell surface (Figure 3A). The various CD3^−^CAR^+^ cells with mutated CD28-TMD were examined for their ability to proliferate to anti-CD28 stimulation. For the analysis of these experiments, CD3^−^CAR^+^ cells were further defined as low, intermediate, or high CAR expression based on the level of the mCherry expression. CAR T cells with the wild-type CD28-TMD (CD28-TMD^WT^) proliferated to anti-CD3/CD28 stimulation, regardless of the level of CAR expression (Figures 3B,C). The CD28-TMD^M4^, but not the other TMD-mutants, abrogated the proliferation of CD3^−^CAR^low^ cells and significantly reduced the proliferation of CD3^−^CAR^int^ cells with either CD8-HD or IgG_4_-HD (Figures 3B,C). Interestingly, CD3^−^CAR^high^ T-cell proliferation was only weakly affected by M4 mutations. The CD3^−^CAR^high^ T cells did not undergo proliferation when restimulation was not carried out, demonstrating that the activation was dependent on anti-CD28 stimulation and was not a result of autonomous CAR tonic signaling (Figure 3B).

To confirm that the CD28-TMD^M4^ disrupted the interaction between CD28 and the CAR, we sorted CAR T-cells based on the Cherry expression, engineered either with CD8-HD/CD28-TMD^WT^ or with CD8-HD/CD28-TMD^M4^, and rechallenged them with a plate-bound anti-CD28 (Figure 3D). In this assay, only CAR T cells with a CD28-TMD^WT^ showed significant proliferation, as measured by radiolabeled-thymidine incorporation. Importantly, co-immunoprecipitation of the endogenous CD28 and the CD28-TMD-containing CAR was abrogated by the M4-mutant, demonstrating that the four amino acids present at the core of the CD28-TMD are necessary for CAR-CD28 heterodimerization (Figure 3E).

### CD28-CAR Heterodimers Response to CD80 and CD86

**Figure 4:**
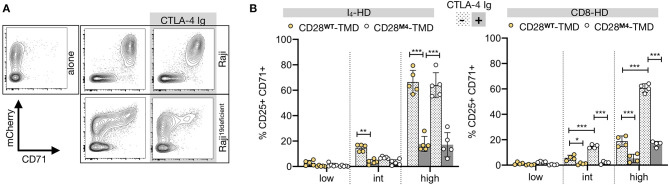
CAR-CD28 heterodimers are B7-unresponsive. **(A)** A representative example showing CD71 upregulation in CAR T cells containing an IgG_4_-HD/CD28-TMD co-cultured for 48 h with irradiated (4,000 Rad) CD19-wild type or deficient Raji cells with or without CTLA-4 Ig. **(B)** CD25^+^CD71^+^ T cells were analyzed in low, intermediate (int), or high mCherry-expressing CAR T cells using the gating strategy described in Supplementary Figure 5. Data were pooled from four independent experiments using T cells from four to five unrelated donors. The two-way ANOVA was used for statistical analysis. *p < 0.05, **p < 0.01, ***p < 0.001.

To determine if the natural ligands of CD28, CD80, and CD86 can activate CARs by engaging CD28-CAR heterodimers, we stimulated CAR T cells with different HD and TMD with CD19-deficient Raji cells that express high levels of CD80 and CD86 (Supplementary Figure 5A). CD19^deficient^ Raji induced CAR T-cell activation, although at a lower intensity than that induced by the CD19^+^ Raji cells (Supplementary Figure 5B, Figure 4A). This “off-target” activation was mostly seen in T cells with a high CAR expression (Figures 4A,B). Moreover, CAR T-cell activation by CD19^deficient^ Raji was significantly reduced by binding CTLA-4 Ig, a high-affinity competitive inhibitor of CD28, to CD80 and CD86 (Figures 4A,B), demonstrating that the off-target activation of CAR T cells is predominantly driven by the CD28 interaction between CD80 and CD86. Importantly, CD28-TMD^M4^ did not markedly change the off-target activation of either IgG_4_-HD/CD28^WT^ or CD8-HD/CD28^WT^ CARs, demonstrating the inability of CD28-CAR heterodimers to respond to natural CD28 ligands.

### Regulation of CD28 Expression by CD28-CAR Heterodimers

**Figure 5:**
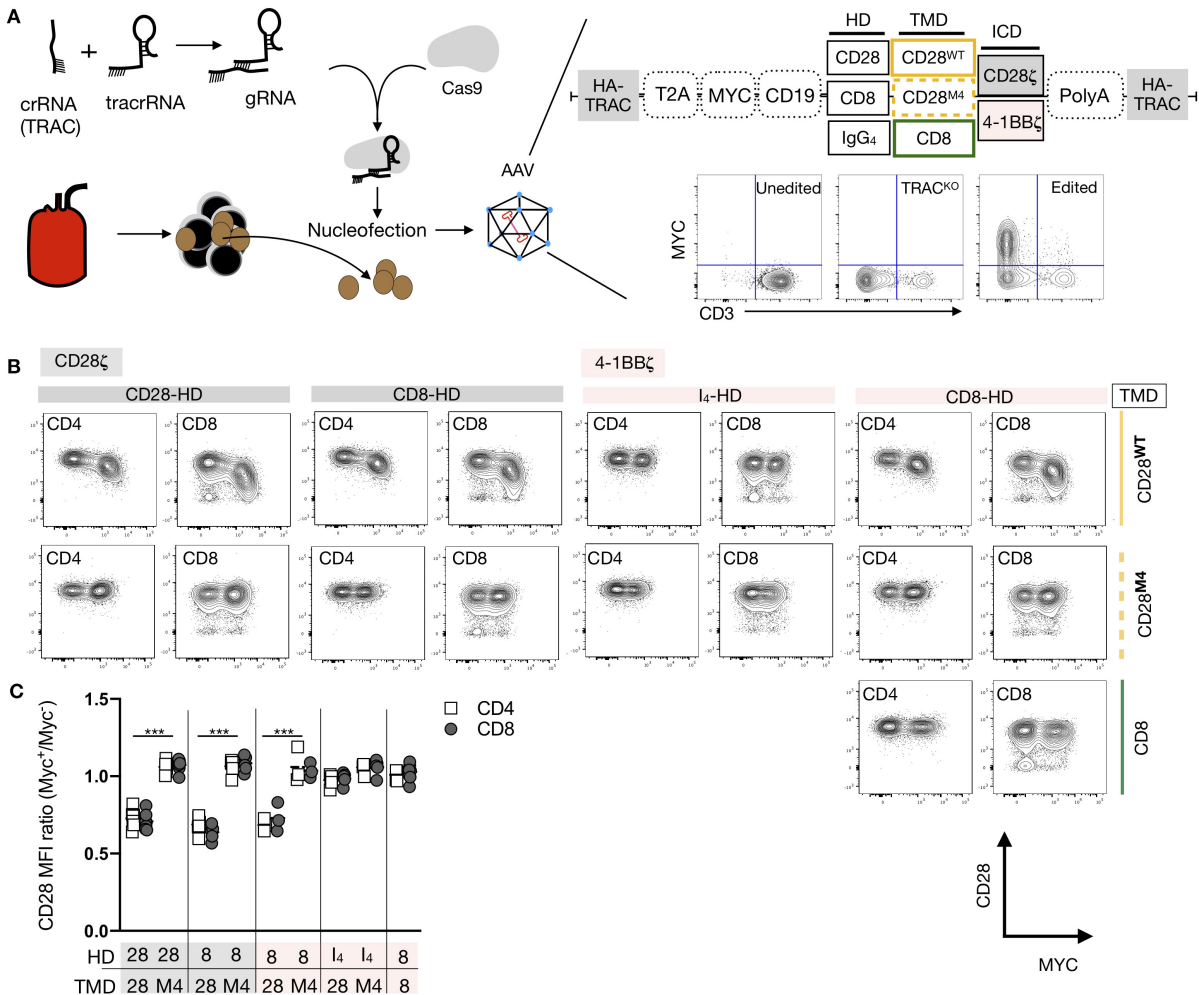
CAR-CD28 heterodimers reduce CD28 expression. **(A)** Editing strategy and homology-directed repair-mediated integration into the TRAC locus of various CD19 CARs using an AAV-6 transduction protocol. **(B)** The expression of Myc and CD28 in a representative example was analyzed 6 days after editing and the removal of beads. **(C)** The CD28 MFI ratio was calculated by dividing CD28 MFI of Myc^+^ cells by Myc^−^ cells in the same culture. Pooled data from three to four independent experiments across five unrelated donors are shown. Each dot represents one independent editing condition. The two-way ANOVA was used for statistical analysis. ***p < 0.001.

We next examined the impact of CAR-CD28 heterodimerization on CD28 expression. Since lentiviral transduction resulted in a wide range of CAR expression levels that influenced on- and off-target T-cell activation, we expressed various CARs by knocking them into the TCR alpha constant (*TRAC*) gene locus using homology-directed repair that provided more homogenous expression of the CAR (Figure 5A) (28). Knock-in efficiencies ranged between 17 and 72% across the various CAR constructs, but the levels of the CAR expression were similar regardless of the differences in editing efficiency (Supplementary Figure 6A). In addition, all CAR T cells proliferated upon stimulation with CD19^+^ NALM-6 target cells (Supplementary Figure 6B), demonstrating that CARs containing M4 mutations remained functional. Six days after CAR knock-in prior to exposing the cells to target cells, we observed a 26–51% reduction in the CD28 mean fluorescence intensity on CAR^+^ T cells containing a wild-type CD28-TMD, but not an M4 CD28-TMD with either a CD8-HD or CD28-HD (Figures 5B,C). This reduction was seen in both CD4^+^ and CD8^+^ T cells with CARs containing either 28**ζ** or 4-1BB**ζ** ICD. The downregulation of CD28 in CAR T cells engineered with an IgG_4_-HD/CD28^WT^-TMD was minimal, echoing the earlier result of inefficient CAR-CD28 heterodimerization in the context of IgG_4_-HD.

### Modeling of Hinge-Hinge Interactions

**Figure 6:**
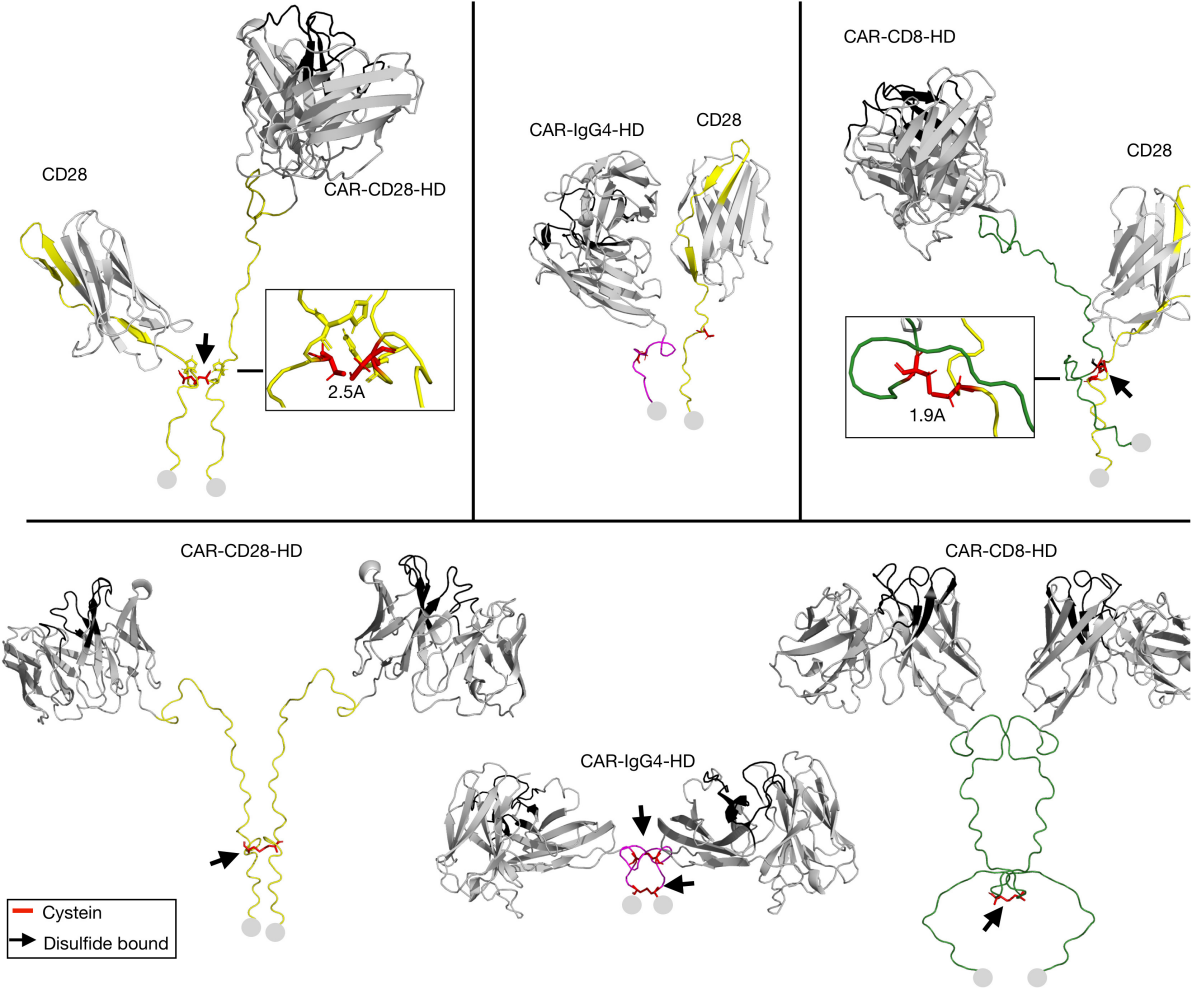
Modeling of hinge-hinge interactions. The extracellular part of the CD28 receptor and CARs engineered with a CD28, IgG_4_, or CD8 HD were modeled as CD28-CAR heterodimers (top) or as CAR-CAR homodimers (bottom). Black arrows point to locations of possible disulfide bonds in the HD. Disulfide bonds are depicted in red. The start of the transmembrane domain is represented by gray circles.

Given the impact of the HD on CAR-CD28 heterodimerization, we next modeled the hinge-hinge interactions to better understand how the HD might influence the interaction of CD28 with CARs. As few TMD templates are available for modeling and their structures difficult to solve by nuclear magnetic resonance spectroscopy, we limited the modeling only to the extracellular domain of the CAR and CD28 receptors. The cysteine residue in the HD of the CD28 receptor C123 was aligned with the cysteine in the HD of CD28-HD-containing and CD8-HD-containing CARs (Figure 6). However, for IgG_4_-HD-containing CARs, the cysteines in the HD could not be aligned with C123 of the CD28 (Figure 6). The modeling presented in the study demonstrated that the presence of a disulfide bound with endogenous CD28-HD is possible for the CD28-HD- and CD8-HD-containing CARs (Figure 6). However, as seen in Figures 5B,C, the lack of CD28 downregulation in CAR T cells with a CD28-HD and a M4-CD28-TMD suggests the CD28-HD alone was not sufficient to mediate the heterodimerization. Moreover, when various CD3^−^CAR^+^ T cells were stimulated with anti-CD3/CD28 beads, we did not observe enhanced CFSE dilution of T cells engineered with the CD28-HD/M4-CD28-TMD CAR construct when compared to CAR T cells with the CD8-HD/M4-CD28-TMD constructs (Supplementary Figures 7A,B). These results further support the notion that the cysteine bridge in the CD28-HD is insufficient to mediate CD28-CAR heterodimerization without interactions in the CD28-TMD. Taken together, these data suggest that cysteines and inter-molecular disulfide bonds in HDs are not the drivers of CAR-CD28 heterodimerization but can be involved in the stabilization of the CAR-CD28 heterodimers.

## Discussion

In this study, we discovered that the CD28-TMD mediates CAR and CD28 heterodimerization *via* a core of up to four polar amino acids. The efficiency of CAR-CD28 heterodimerization depends on the HD but not the ICD. While the heterodimers are unresponsive to CD28 ligands, namely CD80 and CD86, they lead to the downregulation of CD28 on the surface of CAR T cells. These data unveil a new attribute of CD28-TMD that may impact the function of CD28 TMD-containing CAR T cells.

Our results demonstrate that CAR-CD28 heterodimers can be expressed at the cell surface as a consequence of CD28 TMD dimerization. While concluding this study, Leddon et al. reported that CD28 homodimerization was dependent on the YxxxxT motif of the CD28-TMD (29). This motif is shared with the CTLA-4 receptor and is also structurally related to CD3ζ dimerization (30). The CD28 homodimer is covalently linked by a disulfide bond (C123) in the HD (22, 31). Interestingly, CD28 dimerization and its subsequent cell surface expression could be efficiently prevented only upon combined mutations of C123S (in the CD28-HD) and YT/LL (in the CD28-TMD) (29, 31). This demonstrates that the HD is also a critical aspect to consider in the formation of receptor dimers. The results in the study demonstrated the role of HD in CAR-CD28 heterodimerization, with IgG_4_ HD being less efficient than HDs from CD28 and CD8 (32). The hinge-hinge modeling suggests that the membrane proximity of the cysteine in the IgG_4_-HD may not readily form disulfide bonds with the cysteine in the CD28-HD of the endogenous CD28 receptor and therefore leads to preferential CAR-homodimerization (31). This observation may also be linked to inflexibility of the short IgG_4_-HD, leading to steric hindrance by the globular scFv domain (32).

The modeling data suggest that the CD28 extracellular hinge can form a cysteine bridge with the endogenous CD28 receptor. However, the cysteine bridge by itself is likely insufficient to mediate CD28 dimerization. This finding is reported due to high CD28 expression (Figures 5B,C) and the lack of preferential proliferation of T cells engineered with the CD28-HD/M4-CD28-TMD CAR construct (Supplementary Figure 7). These results are consistent with the observation of Leddon et al. (29), who demonstrated that mutations in the CD28-TMD alone strongly reduce the expression of the CD28 receptor. Asimilar reduction in the CD28 expression was found when mutating the C123S in the CD28-HD, further suggesting that the hinge is necessary for CD28 homodimerization, possibly through stabilization of the dimer rather than its formation (29).

The findings that anti-CD28 can activate CAR T cells through the CAR-CD28 heterodimers raised the concern that the natural ligand of CD28 may also induce off-target activation of CAR T cells. The results of the study showed that CAR T cells can indeed be activated in the absence of target antigen in a CD80- and CD86-dependent manner in T cells expressing a high amount of CAR, but this off-target activation was independent of CD28-CAR heterodimers. This may be explained by the fact that CD80 and CD86 are engaging CD28 homodimers (31). It is also possible that the extracellular conformation of the CD28 monomers is not stable enough to interact with CD80/CD86. Nonetheless, these data suggest that the off-target activation was induced by CD28 homodimers. In T cells with a high level of CAR expression, endogenous CD28 homodimers may induce CAR clustering through membrane compartmentalization. Thus, it has been reported that CD28-mediated costimulation can induce coalescence of membrane microdomains that were enriched for signaling molecules, resulting in an enhanced T-cell activation (33). These results encourage further investigations to explore the utility of costimulation blockade as a new approach to prevent off-target CAR toxicities as antigen-presenting cells are present in virtually all organs and that the CD80^+^/CD86^+^ expression is upregulated during inflammation.

We found that upon efficient CD28-CAR heterodimerization, the level of CD28 expression was significantly reduced in both CD4^+^ and CD8^+^ CAR T cells, possibly because of the recruitment of CD28 into CAR heterodimers. This demonstrates that a CD28-TMD-containing CAR may bind away a substantial fraction of CD28 at the cell surface. It is worth noting that since CD28 in the heterodimers with CAR can bind to anti-CD28 in our functional and immunoprecipitations assays, the reduced MFI is likely due to the loss of expression on the cell surface and not a lack of detection. The functional significance of the loss in the CD28 expression remains to be determined experimentally. A recent study has shown that the CD28 expression in 19-CAR T cells engineered with a CD28-TMD was indeed significantly lower when compared to 19-CAR T cells engineered with a CD8-TMD (34). This correlated with a significantly lower number of CD28-TMD-containing CAR T cells in the peripheral blood 1 month after infusion, suggesting that the reduced CD28 expression might have impaired CAR T-cell persistence. Thus, one could speculate that reduced CD28 expression increases the sensitivity of CAR T cells' to exhaustion or alter their differentiation to effector/memory programs (35).

The present study showed a major biochemical difference between CD28-TMD and CD8-TMD CARS, although the exact functional consequences of this difference remain to be investigated. Several studies demonstrated that 19-CAR T cells engineered with a CD28-HD/TMD have increased sensitivity to low abundant antigens as compared to 19-CAR T cells with a CD8-HD/TMD (11–13). CD28-HD/TMD-containing CARs seem also to be associated with an increased risk of neurotoxicity (4, 36–38) as compared to CD8-HD/TMD-containing CARs (6, 8, 39). A recent report suggested that neurotoxic events can be significantly reduced when replacing the CD28-HD/TMD with a CD8-HD/TMD, independent of the signaling domain (34). Another recent report suggested that severe neurotoxicity observed in clinical trials with 19-CAR T-cell may be due to the presence of CD19^+^ murals cells in the vasculature of the brain (40). With these findings, we hypothesize that the CD28-CAR association may increase CAR sensitivity for ectopically expressed low abundant antigens, such as the CD19 expressed on mural cells, thus demonstrating higher off-tumor activation of CARs. The lack of functional analysis on the consequences of CAR-CD28 heterodimerization remains an important limitation of the present study.

In conclusion, this study shows that the CD28-TMD is not inert and can lead to the formation of CD28-CAR heterodimers. This suggests that, in general, TMDs of CARs can impact CAR association with endogenous proteins, which is a function of the CAR T cell. Thus, optimization of CAR designs should consider TMD-mediated receptor interactions.

## Data Availability Statement

The original contributions presented in the study are included in the article/Supplementary Material, further inquiries can be directed to the corresponding author/s.

## Ethics Statement

Ethical review and approval was not required for the study on human participants in accordance with the local legislation and institutional requirements. The patients/participants provided their written informed consent to participate in this study.

## Author Contributions

YDM and QT: conceptualization and manuscript writing. YDM: formal analysis. QT: funding acquisition. YDM, DPN, LMRF, CR, ZC-W, RBV, and LP: investigation. YDM, DPN, TR, PH, JE, and LP: methodology. QT, JAB, AM, and JAW: resources. QT and JAB: supervision. YDM, QT, DPN, LMRF, CR, AM, JE, TR, FVG, ZC-W, RBV, JAW, and JAB: writing-review and editing. All authors contributed to the article and approved the submitted version.

## Conflict of Interest

A provisional patent on CAR-CD28 heterodimerization has been submitted. JAB and QT are co-founders of Sonoma Biotherapeutics. AM and TR are co-founders of Arsenal Biosciences. AM is also a co-founder of Spotlight Therapeutics. JAB and AM have served as advisors to Juno Therapeutics. AM was a member of the scientific advisory board at PACT Pharma and was an advisor to Trizell. QT, JAB, and AM have received sponsored research support from Juno Therapeutics. AM is cofounder, member of the Boards of Directors and member of Scientific Advisory Boards of Spotlight Therapeutics and Arsenal Biosciences. AM has served as an advisor to Juno Therapeutics, was a member of the scientific advisory board at PACT Pharma and was an advisor to Trizell. AM has received honoraria from Merck, a consulting fee from AlphaSights, and is an investor in and informal advisor to Offline Ventures. AM owns stock in Arsenal Biosciences, Spotlight Therapeutics and PACT Pharma. AM has received research support from Epinomics, Sanofi, GlaxoSmithKline, and gifts from Gilead and Anthem. JAW is co-Founder of Soteria Biotherapeutics developing small molecule switchable biologics, on the SAB of Spotlight, and recipient of sponsored research from Bristol Myers Squibb. JE is an advisor for Mnemo Thérapeutics and Cytovia and received research support from Cytovia. TLR is a co-founder, member of Scientific Advisory Board, and founding Chief Scientific Officer of Arsenal Biosciences. The remaining authors declare that the research was conducted in the absence of any commercial or financial relationships that could be construed as a potential conflict of interest.

## Abbreviations

CAR: Chimeric antigen receptor
HD: Hinge domain
ICD: Intracellular signaling domain
scFV: Single chain variable fragment
TMD: Transmembrane domain.
