# Supplementary material for: The CD28-Transmembrane Domain Mediates Chimeric Antigen Receptor Heterodimerization With CD28
Source: Front Immunol. 2021 Mar 23;12:639818. doi: 10.3389/fimmu.2021.639818 (PMC8021955; doi:10.3389/fimmu.2021.639818)
Supplement: Supplementary file 1 [file Data_Sheet_1.docx]

Supplementary Material

# Supplementary Figures


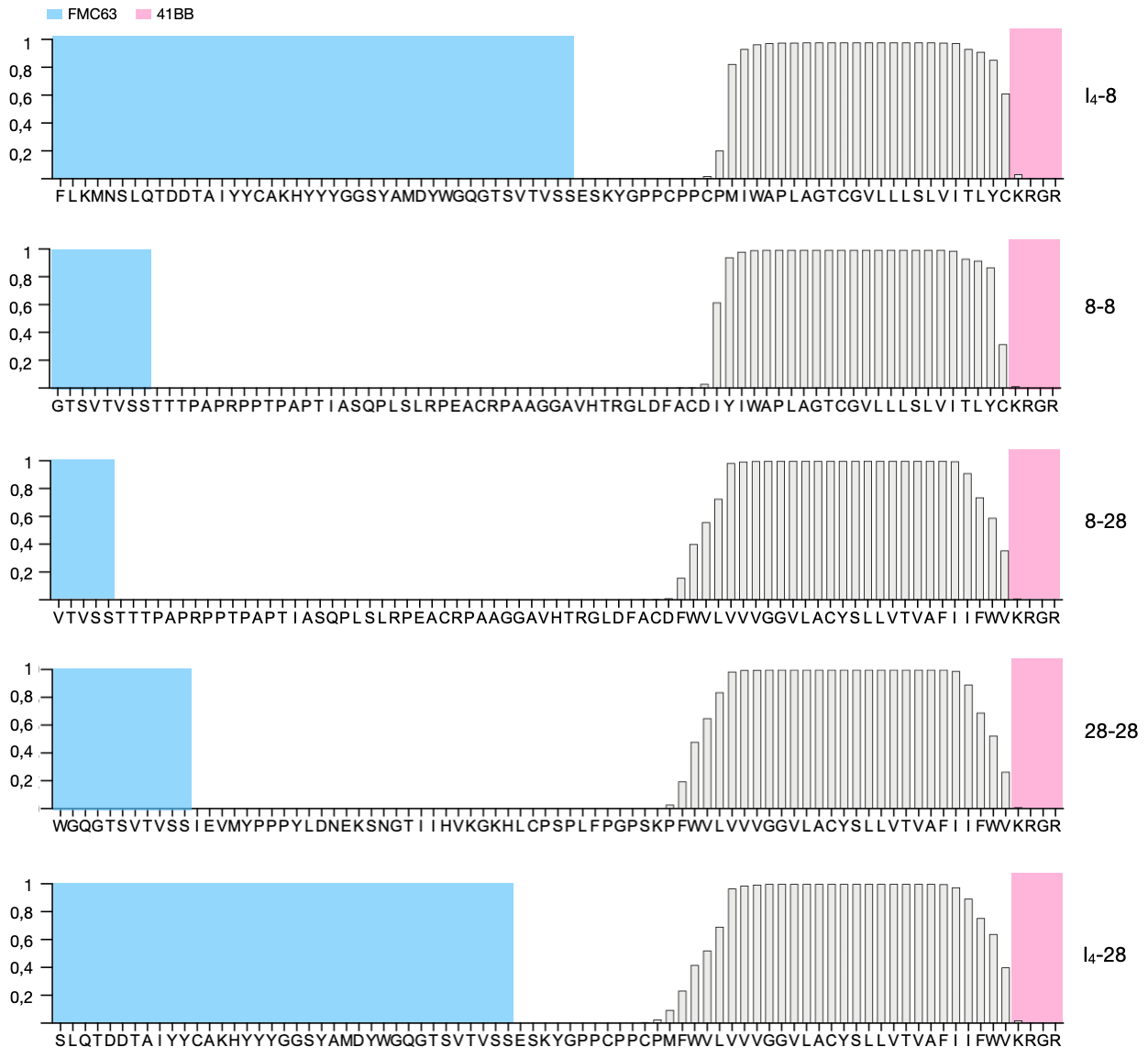


**Supplementary Figure 1.** Sequences of five CD19-CAR constructs differing by their hinge and transmembrane domains.

The anti-CD19 single chain variable fragment (ScFv) is shown in a light blue shade. Transmembrane predictions are shown as light grey bars and the 4-1BB intracellular domains are shown in a pink shade. Transmembrane probability (height of grey bars) was determined using an online tool (<http://www.cbs.dtu.dk/services/TMHMM/>) based on a hidden Markov model ^41^.

.


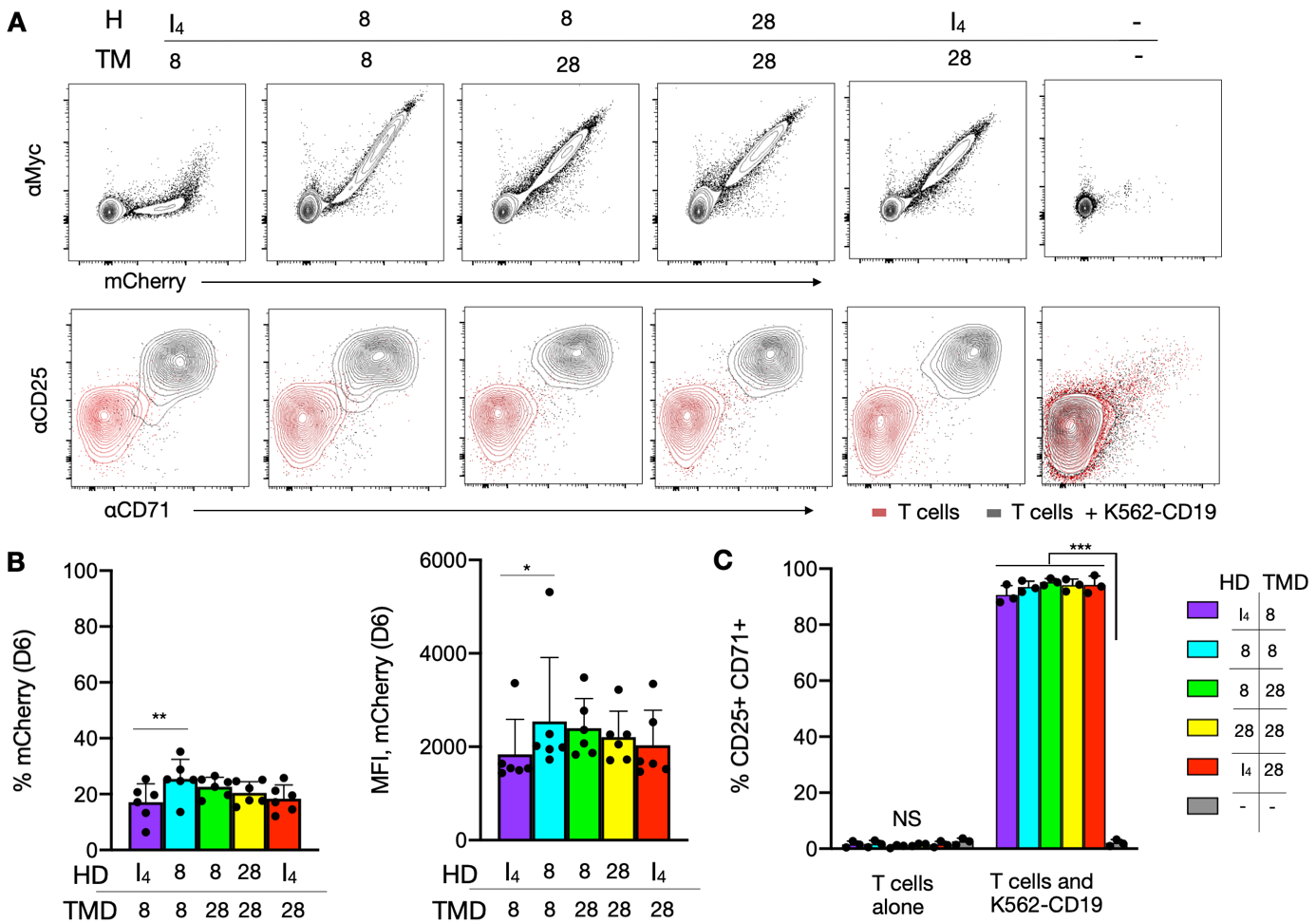


**Supplementary Figure 2.** Anti-CD19 CAR expression on CD4 T cells

**(A**) Top row: representative flow cytometric analysis of the 5 different CAR constructs showing the transduction profile of Cherry^+^ and Myc^+^ among CD4^+^ T cells. Bottom row: flow dot plot overlays of T-cell activation (measured by CD25 and CD71 expression) when cultured with (gray) or without (red) CD19-expressing K562 (K562-CD19) cells. **(B)** A summary of the percentage of mCherry^+^ cells and mCherry MFI (gated on mCherry positive cells) from 3 independent experiments using T cells from 6 independent donors is shown. One-way ANOVA was used for statistical analysis. (**C**) CD25^+^ CD71^+^ expression among CD4^+^ T cells was analyzed by comparing T cells cultured alone or with CD19-expressing K562 cells. A summary of the results from 2 independent experiments using T cells from 3 independent donors is graphed. Two- way ANOVA analysis was used for statistical analysis, * p<0.05, ** p<0.01, *** p<0.001.s

*
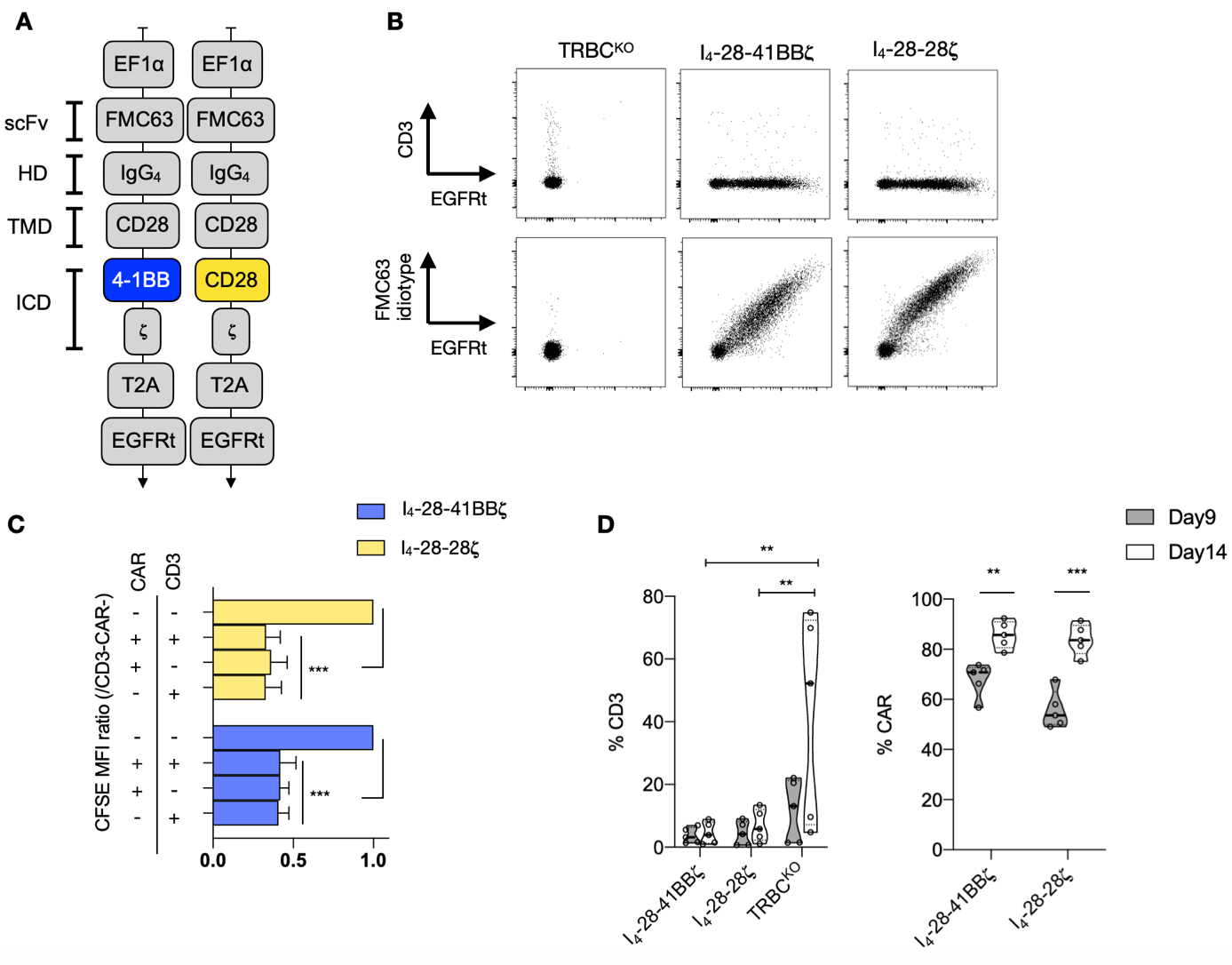
*

**Supplementary Figure 3.** Proliferation of CD19-CAR T cells expressing a 28𝛇 or 41bb𝛇 intracellular domain

**(A)** Construct design of CARs bearing an IgG_4_ hinge, CD28 transmembrane (TMD) domain with either a CD28 CD3𝛇 or 4-1BB CD3𝛇 intracellular domain (ICD). Both CARs are fused to a T2A-EGFRt reporter. (**B**) Representative plots illustrating editing efficiency 6 days after transduction. (**C**) On day 9 of culture, a mixed population of CD3^+/-^CAR^+/-^ cells was labeled with CFSE and re-stimulated with anti-CD3/CD28 beads and cultured for 4 days. Normalized CFSE MFI for CD3^+^EGFRt^-^, CD3^+^EGFRt^+^ and CD3^-^ CAR^+^ cells were calculated by dividing CFSE MFI of these populations with the MFI of the CD3^-^CAR^-^ cells in the same culture. Results from 4-6 independent donors from 3 independent experiments are summarized. One-way ANOVA was used for statistical analysis. (**D**) On day 9 of culture, a mixed population of CD3^+/-^CAR^+/-^ cells was re-stimulated with anti-CD3/CD28 beads and IL-2 (30 IU/mL) and expanded for another 5 days. The percentage of CD3 and CAR expression was compared before (D9) and after restimulation (D14) by flow cytometry. Results from 5 independent donors from 3 independent experiments are summarized. ** p<0.01, *** p<0.001.


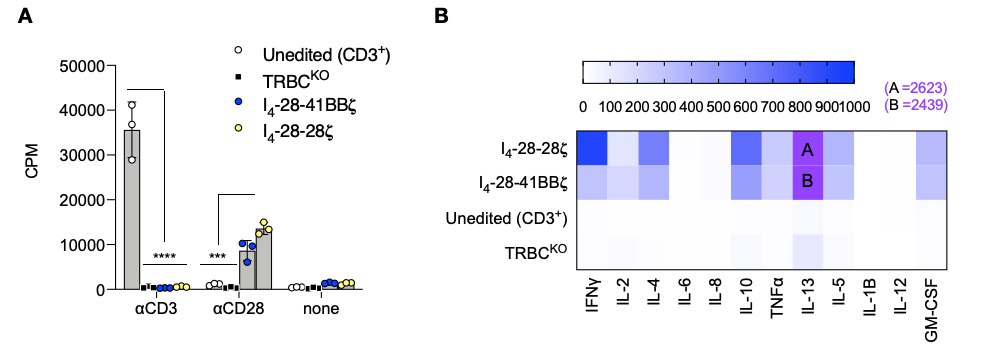


**Supplementary Figure 4.** Anti-CD28 dependent proliferation and cytokine production of CD19-CAR T cells expressing a 28𝛇 or 41BB𝛇 intracellular domain

(**A**) CD3^-^CD4^+^ T cells expressing CD19-CAR engineered with an IgG_4_-HD/CD28-TMD-28𝛇-ICD or IgG_4_-HD/28-TMD-4-1BB𝛇-ICD CAR were FACS purified based on EGFRt expression. Cells were stimulated with soluble anti-CD28 (clone CD28.2, 1 µg/mL), soluble anti-CD3 (clone HIT3α, 2µg/mL), or no stimulation. Proliferation was assessed using ^3^H-thymidine incorporation 64 hours later, with ^3^H thymidine added during the last 16-18 hours. One-way ANOVA was used for statistical analysis. Representative results of 4-7 independent experiments for each condition are shown. One-way ANOVA was used for statistical analysis. (**B**) Cytokines secretion during the first 48 hours after stimulation with soluble anti-CD28 (clone CD28.2, 1 µg/mL) was measured using multiplexed Luminex. Results units are pg/mL and are a summary of 4-7 independent experiments using T cells from 4 independent donors. *** p<0.001.


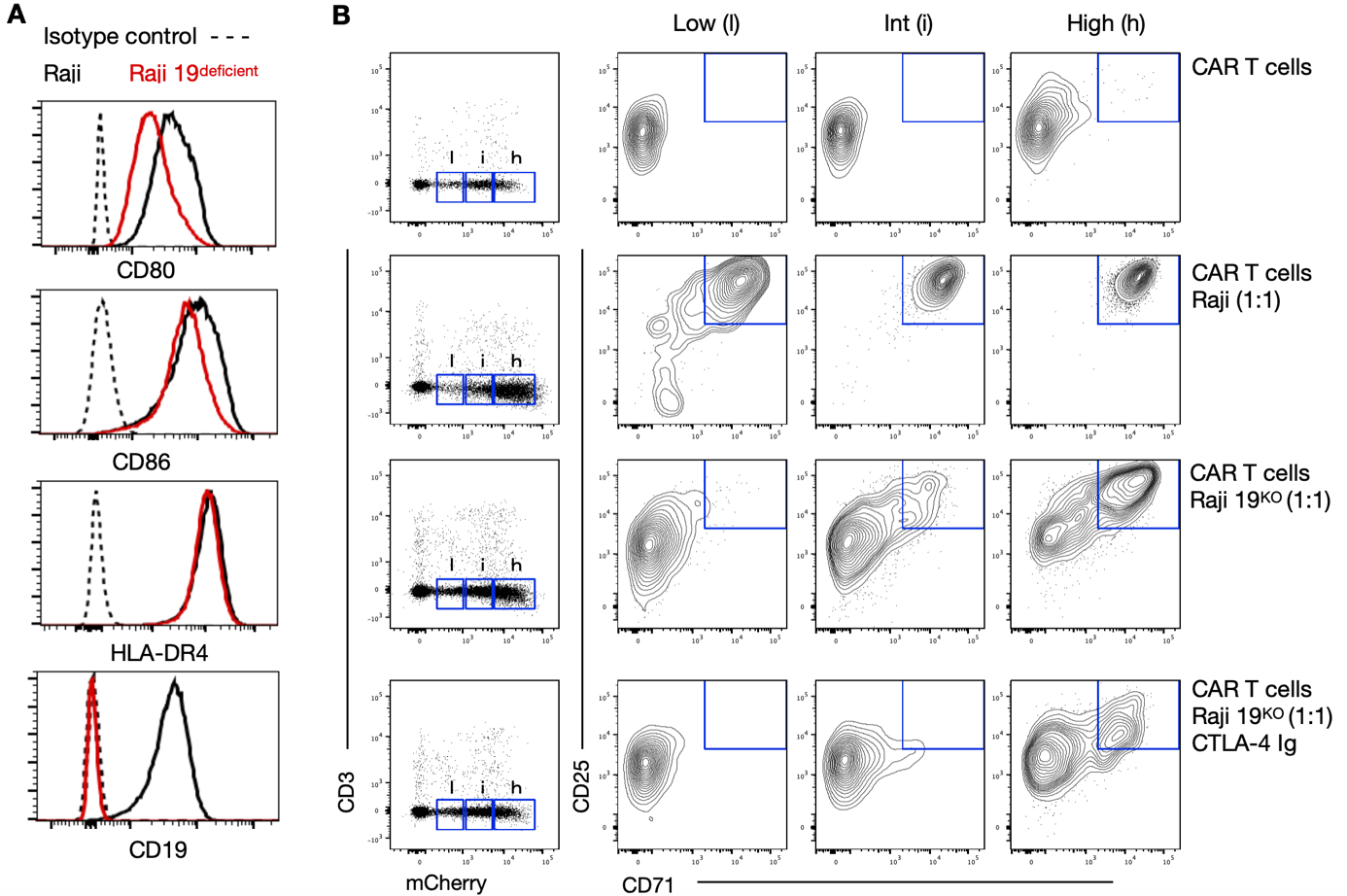


**Supplementary Figure 5.** Mixed lymphocyte reaction with CD19-deficient Raji cells.

(**A**) Representative staining for CD80, CD86, HLA-DR4 and CD19 of Raji cells. (**B**) CAR T cells were co-cultured alone, with wild type or with CD19-deficient irradiated Raji (4000 rad) cells at 1:1 ratio with or without CTLA-4 Ig. Gating strategy used to define the percentage of CD25^+^CD71^+^ expression among the mCherry low (l), intermediate (i), and high (h) CD4+CD3- cells.


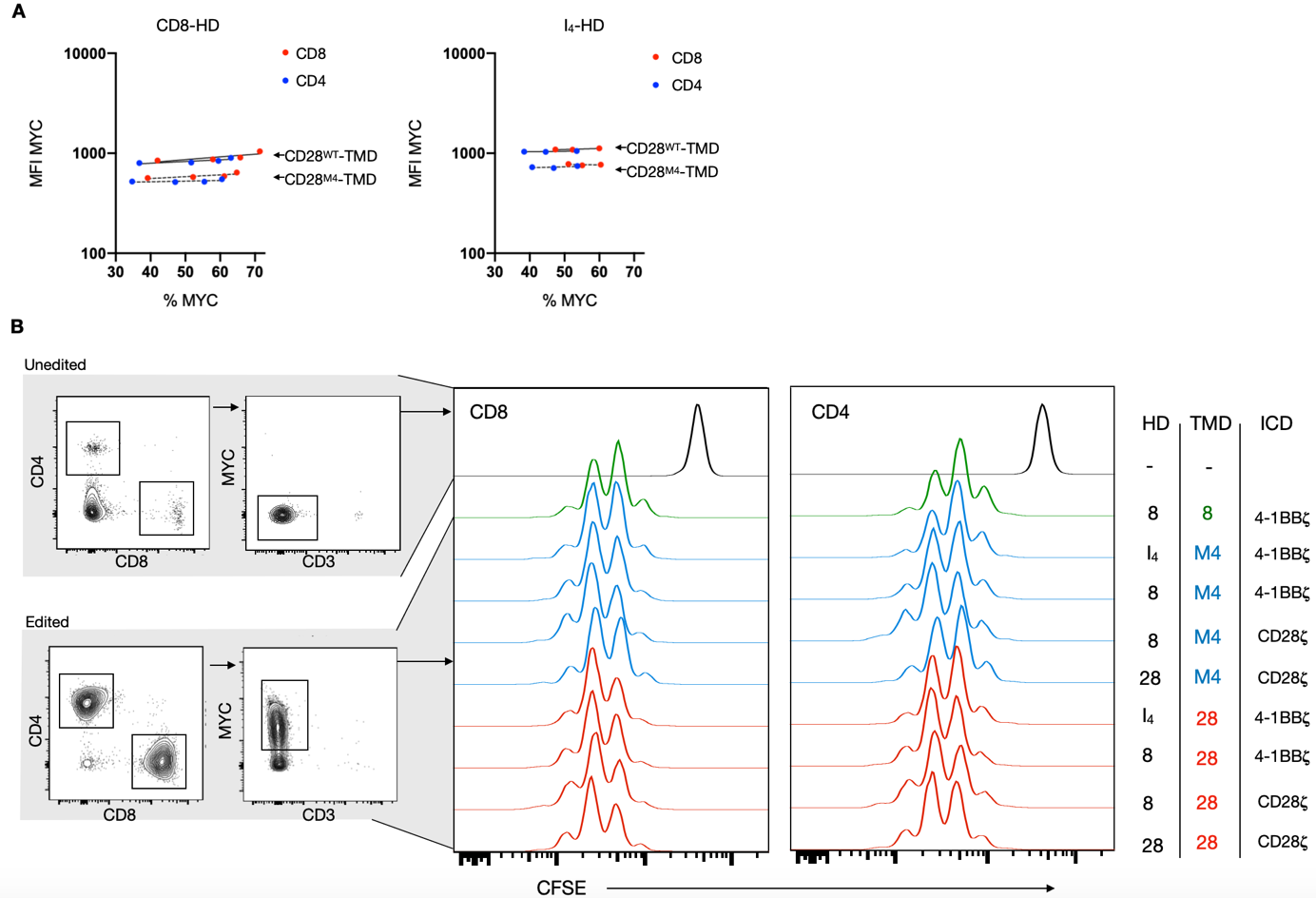


**Supplementary Figure 6.** AAV-transduced CAR expression and proliferation

(**A**) Transduction efficiency was defined by the percentage of MYC^+^CD3^-^ cells. Following electroporation, AAV6 viruses were tittered resulting in different transduction efficiencies. MYC MFI was defined for each condition. The titration for the CAR constructs bearing a CD8-HD or an IgG_4_-HD were performed on two separate experiments with two independent donors. (**B**) On day 8 of culture, edited and unedited T cells were stained for CFSE and co-cultured with NALM-6 cells for 4 days. The gating strategy and CFSE dilutions are shown. A representative example of two independent experiments is shown.


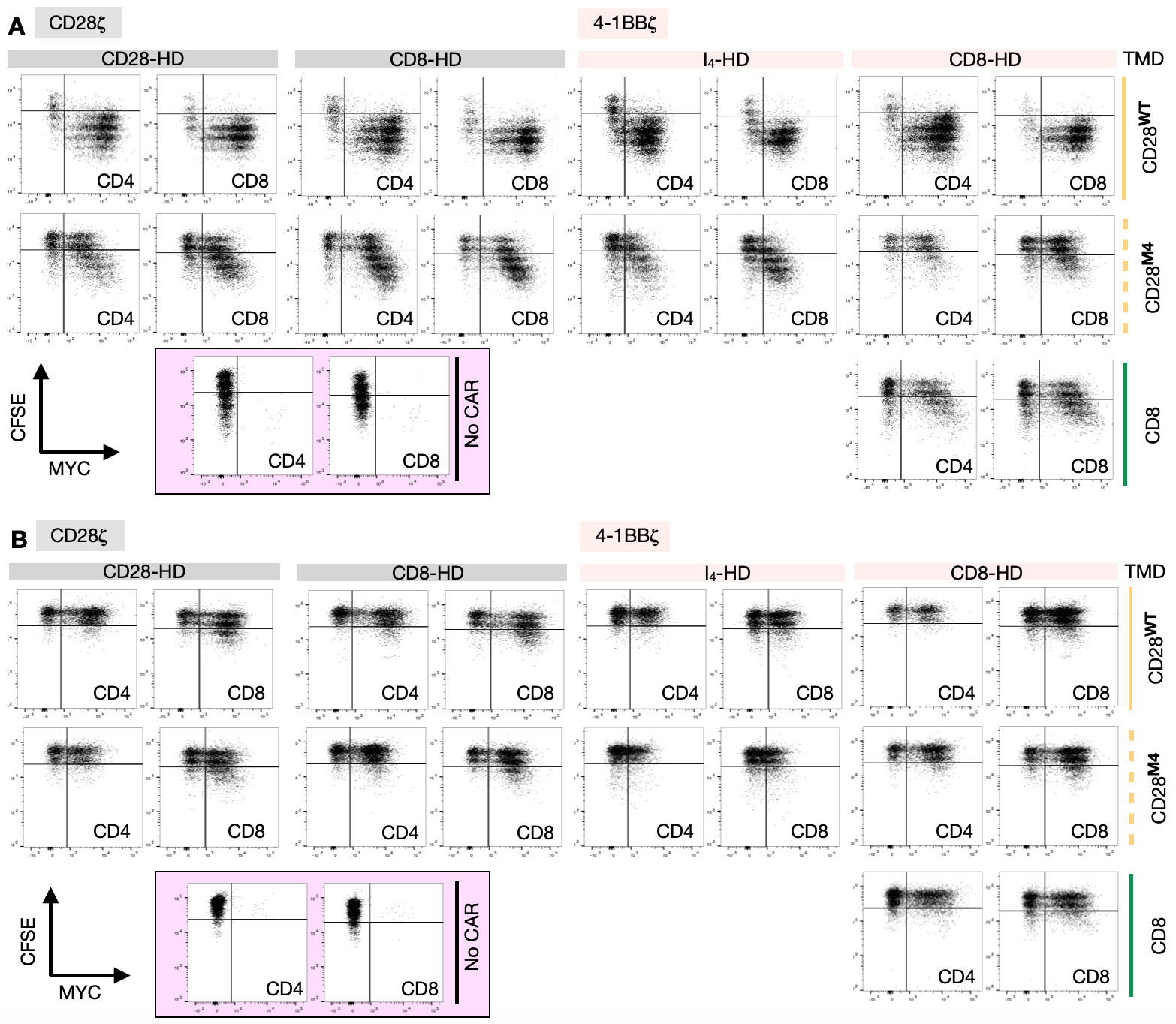


**Supplemental Figure 7.** Proliferation of CAR T cells.

A representative example from two independent experiments of CFSE dilution of CD3-CAR+ T cells re-stimulated with anti-CD3/28 beads (A) or left unstimulated (B).
